# Neuroprotection of Tanshinone IIA against Cerebral Ischemia/Reperfusion Injury through Inhibition of Macrophage Migration Inhibitory Factor in Rats

**DOI:** 10.1371/journal.pone.0040165

**Published:** 2012-06-29

**Authors:** Yanlin Chen, Xuemei Wu, Shanshan Yu, Xuemei Lin, Jingxian Wu, Lan Li, Jing Zhao, Yong Zhao

**Affiliations:** 1 Department of Pathology, Chongqing Medical University, Chongqing, People’s Republic of China; 2 Department of Pathophysiology, Chongqing Medical University, Chongqing, People’s Republic of China; The Chinese University of Hong Kong, Hong Kong

## Abstract

**Background:**

Ischemia/reperfusion (I/R) injury is associated with systemic inflammatory response. Macrophage migration inhibitory factor (MIF) has been implicated in many inflammatory processes. Tanshinone IIA (TSA) is one of the active ingredients in danshen, which derived from the dried root or rhizome of *Salviae miltiorrhizae Bge*. Recent studies have demonstrated that TSA has protective effects against focal cerebral I/R injury. However, little is known about the underlying mechanisms. Here we put forward the hypothesis that TSA acts through inhibition of MIF expression during focal cerebral I/R injury in rats.

**Methodology/Principal Findings:**

Rats were subjected to middle cerebral artery occlusion (MCAO) for 2 hours. This was followed by reperfusion. We measured neurological deficits, brain water content, and infarct volume, and found that neurological dysfunction, brain edema, and brain infarction were significantly attenuated by TSA 6 hours after reperfusion. We also measured myeloperoxidase (MPO) activity at 6 and 24 hours, and found that neutrophil infiltration was significantly higher in the vehicle+I/R group than in the TSA+I/R group. ELISA demonstrated that TSA could inhibit MIF expression and the release of TNF-α and IL-6 induced by I/R injury. Western blot analysis and immunofluorescence staining showed that MIF expression was significantly lower in the TSA+I/R group than in the vehicle+I/R group. MIF was found almost all located in neurons and hardly any located in astrocytes in the cerebral cortex. Western blot analysis and EMSA demonstrated that NF-κB expression and activity were significantly increased in the vehicle+I/R group. However, these changes were attenuated by TSA.

**Conclusion/Significance:**

Our results suggest that TSA helps alleviate the proinflammatory responses associated with I/R-induced injury and that this neuroprotective effect may occur through down-regulation of MIF expression in neurons.

## Introduction

Stroke is the second most common cause of death and a major cause of disability worldwide [1], [2]. The inflammatory response to brain injury plays a vital role in the pathogenesis of stroke [3], [4]. The selective inhibition of inflammatory cytokine activity remains an important goal in the effective treatment of brain ischemia and reperfusion (I/R) injury. Recent studies have demonstrated that agents with anti-inflammatory action have therapeutic potential in experimental models of stroke [5], [6].

Macrophage migration inhibitory factor (MIF) is a proinflammatory cytokine derived from many cell types [7]. After activating nuclear factor κB (NF-κB), MIF induces the production of subsequent cytokines [8]. There is ample evidence indicating that MIF expression is increased at the transcriptional level in human stroke patients and in animal models of focal ischemia [9]. This suggests that inhibition of MIF may serve as a viable strategy for the treatment of ischemic stroke.

**Figure 1:**
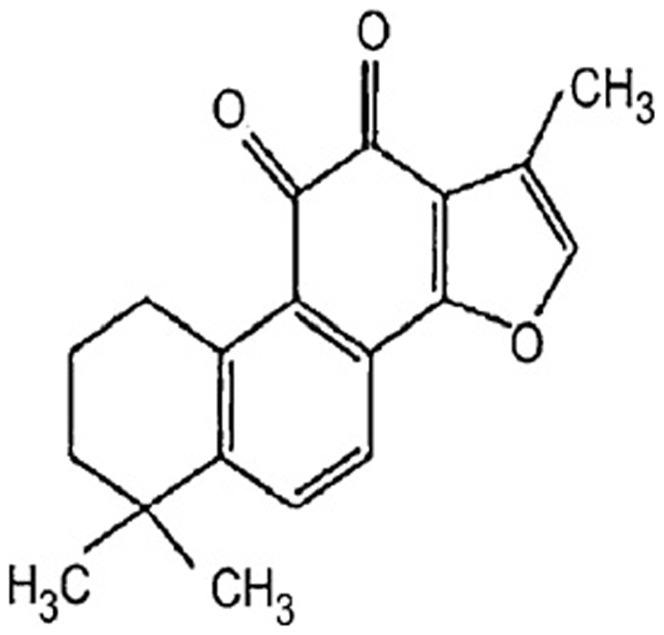
Chemical structure of Tanshinone IIA.

Danshen, a very important component of Chinese medicine derived from the dried root or rhizome of *Salviae miltiorrhizae Bge* (SM), has been widely used in China for the treatment of cerebrovascular conditions, such as ischemic stroke [10], [11]. Tanshinone IIA (TSA), whose IUPAC name is Phenanthro [1, 2-b] furan-10, 11-dione, 6, 7, 8, 9-tetrahydro-1, 6, 6-trimethyl being a derivative of phenanthrenequinone, is a key active component of danshen (Fig. 1) [12]. Recent studies have demonstrated that TSA has protective effects against focal cerebral I/R injury [13], [14]. Our previous study indicated that 25 mg/kg TSA administered 10 minutes after middle cerebral artery occlusion (MCAO) showed most protective effects [15]. However, little is known about the mechanism responsible for the effects of TSA. Researchers have reported that TSA attenuates seawater-aspiration-induced lung injury by inhibiting MIF [16]. Given the importance of MIF, this study puts forward the hypothesis that the neuroprotective effects of TSA may be associate inhibition of the MIF pathway.

## Results

### Effects of TSA on Neurological Deficit, Brain Water Content, and Infarction

**Figure 2:**
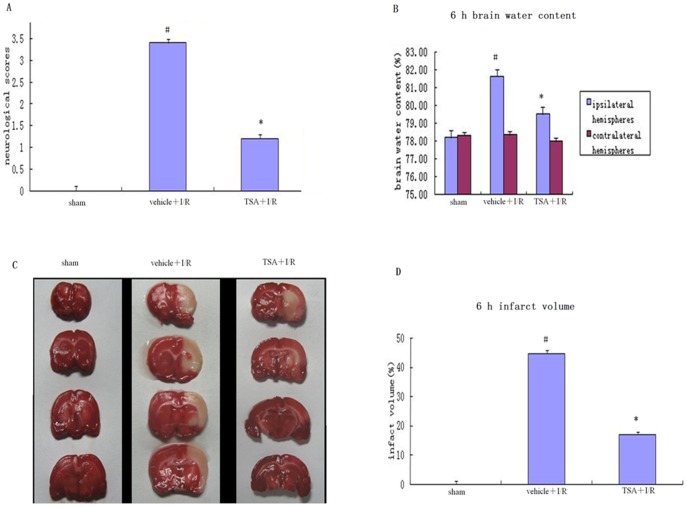
Effects of TSA on neurological deficit, brain water content, and infarction. As shown in Fig. 2, the (A) neurological score and (C and D) infarct volume were significantly higher in the vehicle+I/R group than in the sham group after reperfusion at 6 hours (#*P*<0.05) and lower in the TSA+I/R group than in the vehicle+I/R group (*P<0.05, one-way ANOVA, n = 5–6 for each group). (B) The brain water content of the ipsilateral hemispheres was similar to the neurological score and infarct volume. No difference was found in contralateral hemispheres (*P*>0.05).

To determine the neuroprotective effect of TSA against I/R injury, we measured the neurological score, brain water content, and infarct volume with and without administration of TSA. As shown in Figures 2A, C, and D, relative to the vehicle+I/R group, neurological scores and cerebral infarct volumes were significantly decreased after treatment with TSA (*P*<0.05). As shown in Figure 2B, in the sham group, the brain water content was 78.28±0.16%. In the TSA+I/R group, the brain water content was lower, 79.52±0.21%, than in the vehicle+I/R group 81.64±0.55% (*P*<0.05). No significant differences were observed in contralateral hemispheres (*P*>0.05).

### Effects of TSA on Neutrophil Infiltration in the Brain Tissues

**Figure 3:**
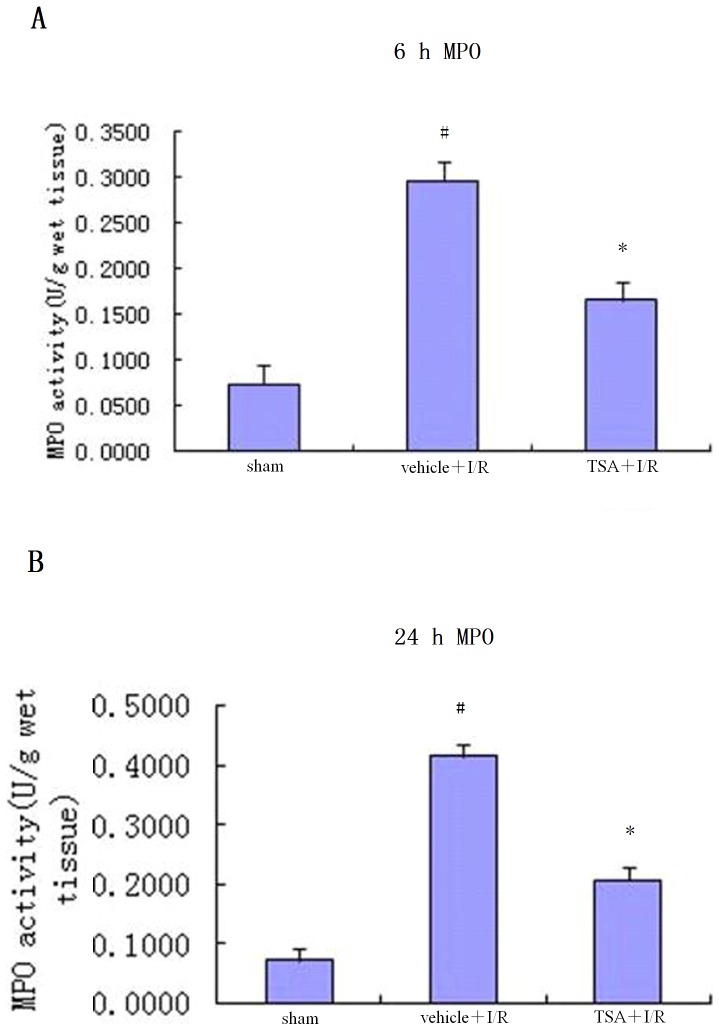
Effects of TSA on MPO activity. MPO activity at (A) 6 hours and (B) 24 hours reflects neutrophil infiltration in the ischemia cerebral cortex. MPO activities were significantly increased in the vehicle+I/R group at the two time points and lower in the TSA+I/R group than in the vehicle+I/R group. Data are mean ± S.E.M., #*P*<0.05 vs. sham group; *P<0.05 vs. vehicle+I/R group.

Next, we performed a myeloperoxidase (MPO) activity assay to determine the neutrophil influx in the ischemia cerebral cortex (Figure 3). MPO activity was significantly higher in the vehicle+I/R group than the sham group at different points in time (*P*<0.05). The increased MPO activity was reduced by treatment with TSA after I/R injury (*P*<0.05).

### Effects of TSA on MIF and Cytokine Expression Induced by Reperfusion at Different Times

**Figure 4:**
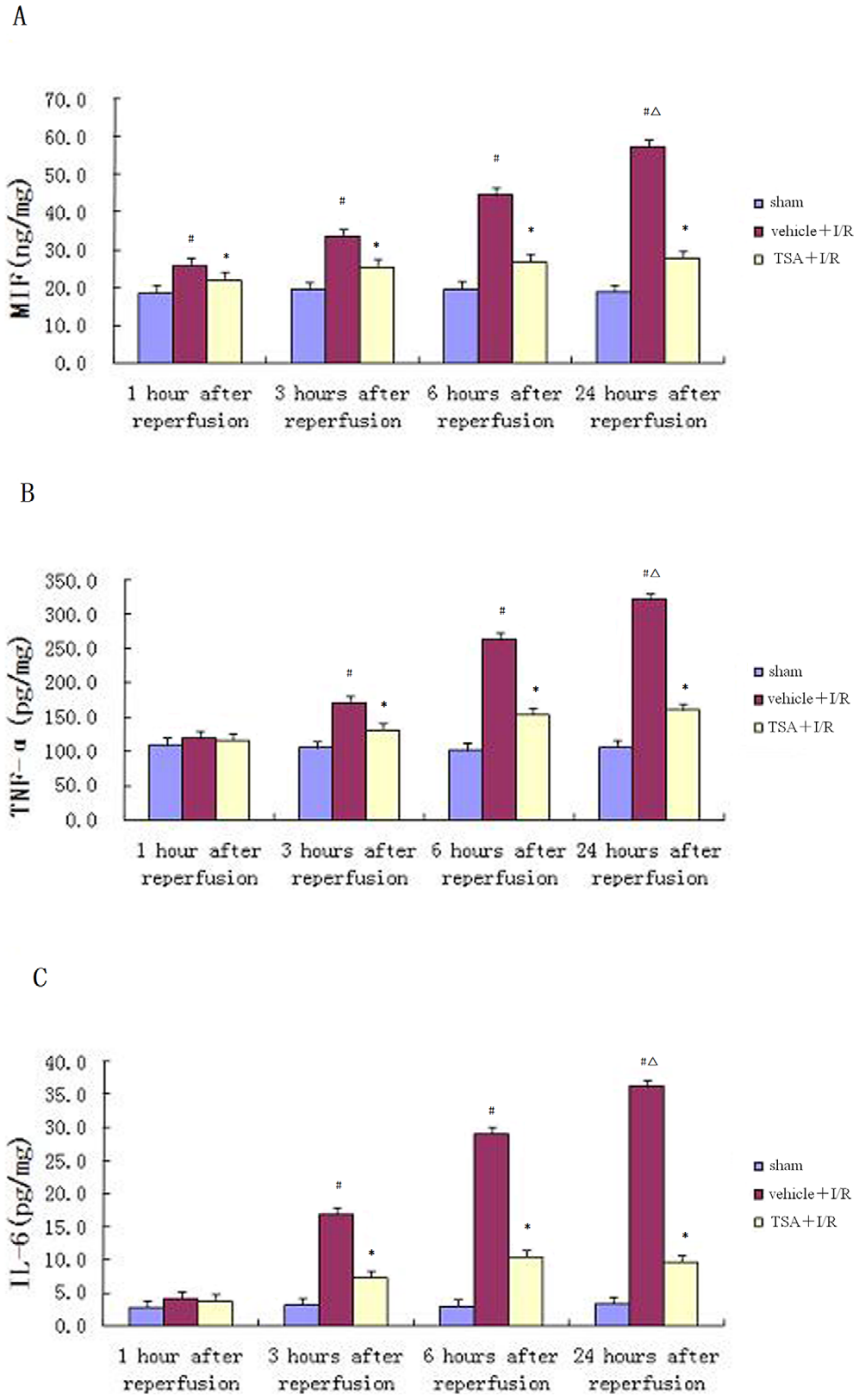
Effects of TSA on expression of proinflammatory cytokines. As shown in Fig. 4A, after reperfusion, MIF content was significantly increased at 1 hour, 3 hours, and 6 hours, reaching a maximum at 24 hours ( Δ*P*<0.05) after reperfusion in vehicle+I/R groups, showing significant differences from sham groups (#*P*<0.05). TSA markedly inhibited the expression of MIF at different points in time after reperfusion (*P<0.05). No visible difference in TNF-α expression had been induced by reperfusion at 1 hour. The elevation of TNF-α level was observed at 3 hours and 6 hours, reaching a maximum at 24 hours (Δ*P*<0.05) after reperfusion in vehicle+I/R groups over sham groups (#*P*<0.05). The change of IL-6 expression was similar to TNF-α level. The increased expression of TNF-α and IL-6 at 3 hours, 6 hours, and 24 hours after reperfusion were also down-regulated by TSA treatment (*P<0.05).

We also examined the effect of TSA on the expression of MIF, tumor necrosis factor-α (TNF-α) and interleukin-6 (IL-6) induced by the reperfusion at different points in time. As shown in Figure 4A, MIF content was significantly higher in the vehicle+I/R group than in the sham group at 1 hour, 3 hours, and 6 hours after reperfusion, showing a maximum difference at 24 hours (*P*<0.05). TSA markedly inhibited the expression of MIF at different points in time after reperfusion (*P*<0.05). No difference in TNF-α expression was observed at 1 hour. The elevation of TNF-α levels was observed 3 hours and 6 hours after reperfusion and found to reach a maximum at 24 hours after reperfusion (*P*<0.05, Figure 4B). The change in IL-6 expression was similar to TNF-α level (Figure 4C). The increased expression of TNF-α and IL-6 at 3 hours, 6 hours, and 24 hours after reperfusion were also down-regulated by TSA treatment (*P*<0.05).

### Effects of TSA on Expression of MIF and NF-κB p65

**Figure 5:**
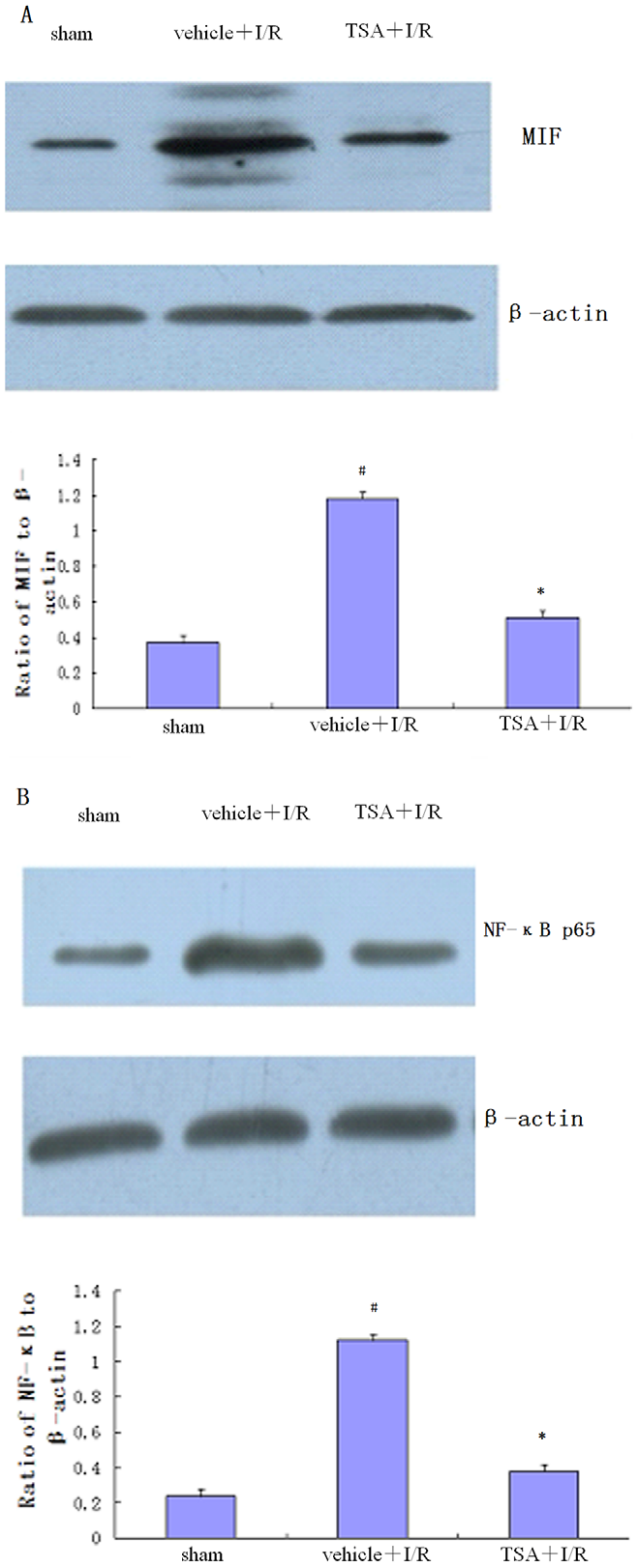
Effects of TSA on expression of MIF and NF-κB p65 24 hours after I/R injury. The protein expressions of (A) MIF and (B) NF-κB p65 in the brain tissues of the injured cerebral hemisphere were detected by Western blot (n = 6 for each group, #*P*<0.05 vs. sham, *P<0.05 vs. vehicle+I/R). After TSA treatment, both the expression of MIF and NF-κB p65 was clearly decreased relative to the vehicle+I/R group.

Western blot analysis (Figure 5) of brain samples showed that the expression level of MIF was increased in the vehicle+I/R group 24 hours after focal cerebral I/R and significantly lower in the TSA treatment group than in the vehicle+I/R group (*P*<0.05). The expression level of NF-κB p65 was also increased in the vehicle+I/R group 24 hours after focal cerebral I/R and significantly lower in the TSA treatment group than in the vehicle+I/R group (*P*<0.05).

### Effects of TSA on NF-κB Activation

**Figure 6:**
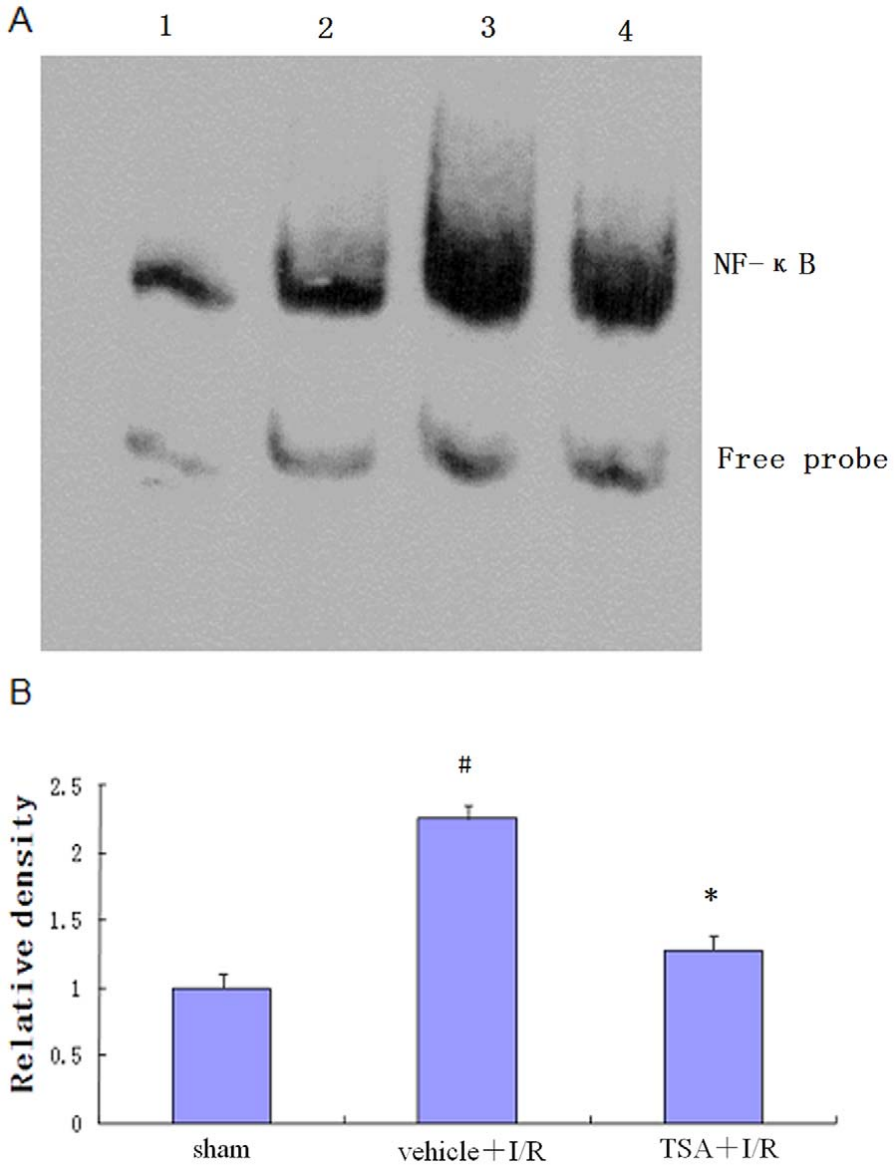
NF-κB activity in the cortex of rat. (A) Nuclear proteins of brain samples of three groups were assayed for NF-κB binding activity by EMSA 24 hours after focal cerebral I/R. Lane 1, positive control; Lane 2, sham group; Lane 3, vehicle+I/R group; Lane 4, TSA+I/R group. (B) Quantification of NF-κB binding activity was performed by densitometric analysis. Bar graph showing the relative density of the EMSA autoradiograph of the three groups compared to sham group. The figure indicates that cerebral NF-κB activity was significantly increased in vehicle+I/R group (#*P*<0.05), which was significantly lower in the TSA treatment group than in the vehicle+I/R group (*P<0.05). Data represents mean ± S.E.M. (n = 5 per group).

NF-κB activation 24 hours after reperfusion was assessed by electrophoretic mobility shift assay (EMSA). As shown in Figure 6, low NF-κB binding activity was observed in sham-operated rats. I/R induced activation of NF-κB in the ipsilateral hemispheres. NF-κB binding activity was increased in the vehicle+I/R group 24 hours after focal cerebral I/R and significantly lower in the TSA treatment group than in the vehicle+I/R group (*P*<0.05).

### Effects of TSA on Location of MIF

**Figure 7:**
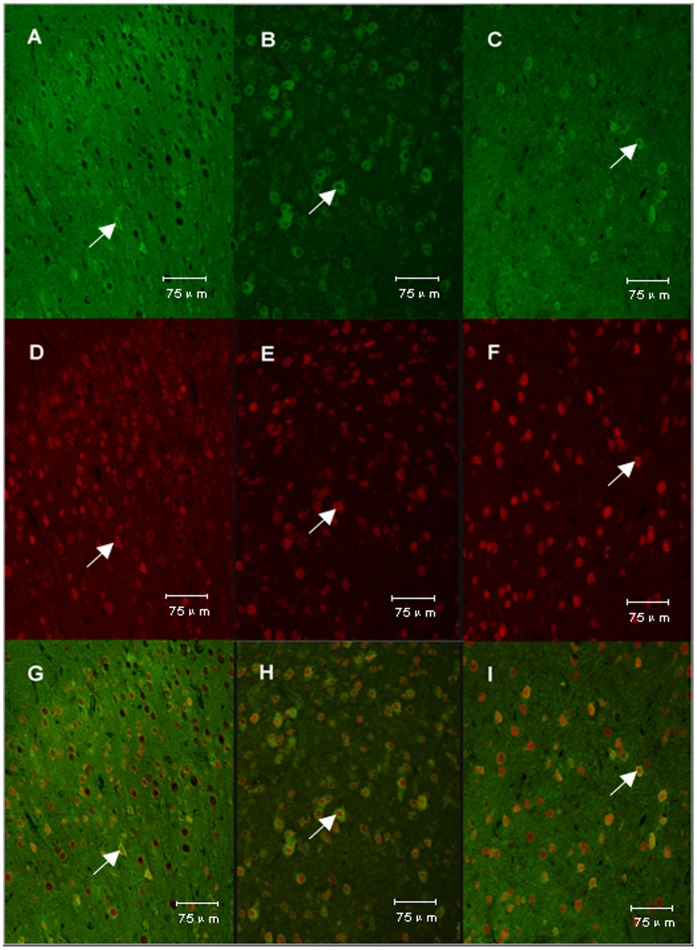
Co-localization of MIF and NeuN. As shown in Fig. 7, A, B and C represent the expression of MIF in sham group, vehicle+I/R group and TSA+I/R group respectively; D, E and F represent the expression of NeuN in sham group, vehicle+I/R group and TSA+I/R group respectively; G, H and I represent the merger of MIF and NeuN in sham group, vehicle+I/R group and TSA+I/R group respectively. Original magnification, ×400. The expression of MIF was increased in the vehicle+I/R group and attenuated by TSA. In the cerebral cortex, MIF was found almost all located in the neurons in the three groups.

**Figure 8:**
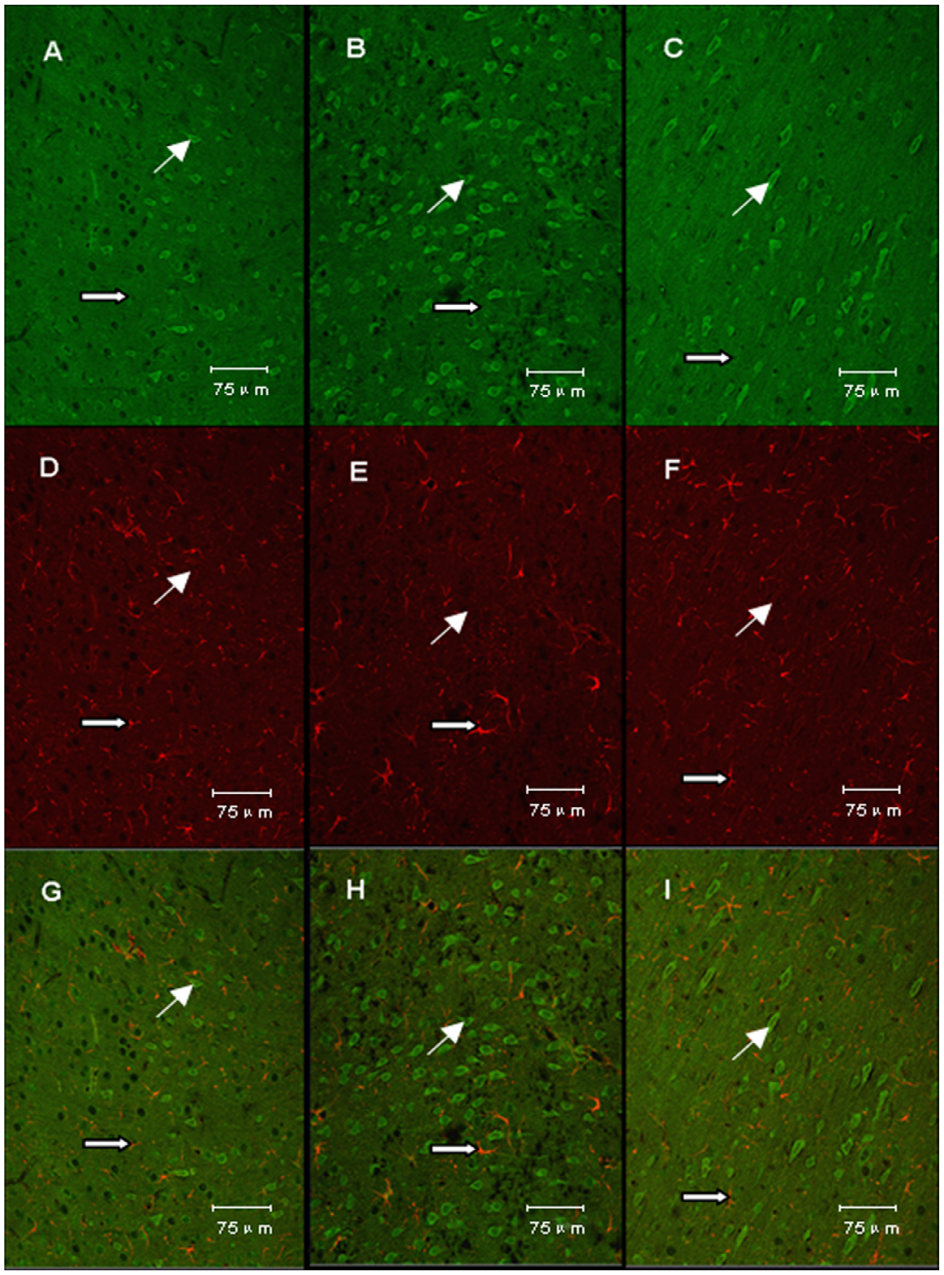
Co-localization of MIF and GFAP. As shown in Fig. 8, A, B and C represent the expression of MIF in sham group, vehicle+I/R group and TSA+I/R group respectively; D, E and F represent the expression of GFAP in sham group, vehicle+I/R group and TSA+I/R group respectively; G, H and I represent the merger of MIF and GFAP in sham group, vehicle+I/R group and TSA+I/R group respectively. Original magnification,×400. The expression of MIF was increased in vehicle+I/R group and attenuated by TSA. In the cerebral cortex, MIF was scarcely located in astrocytes in the three groups.

As shown in Figure 7 and Figure 8, in the cerebral cortex, MIF was found almost all located in neurons and hardly any located in astrocytes. Following surgery, MIF showed significant increases around the infarct core in the vehicle+I/R group and significantly decreases in the TSA+I/R group 24 hours after reperfusion.

## **Discussion**

MCAO is a classical model of cerebral ischemia [17], [18]. In this study, we have shown that TSA can reduce MPO activity after focal cerebral I/R injury. We also demonstrated that TSA can inhibit MIF expression, NF-κB activity and the release of TNF-α and IL-6 induced by I/R injury. TSA was here found to provide neuronal protection partly through its anti-inflammatory effect associated with down-regulation of MIF. Our results also indicated that MIF is presenting almost all located in neurons and rare located in astrocytes.

Danshen, a very important traditional Chinese medicinal herb, has been commonly used in traditional Chinese medicine practice for over a thousand years in the treatment of coronary artery disease and cerebrovascular diseases including stroke. The therapeutic efficacy of Danshen in stroke has been confirmed by clinical studies suggesting that Danshen preparations were neuroprotective with few or no adverse effects [19]. In recent decades, TSA being one of the active ingredients in danshen has been widely used in research for the treatment of cadiovascular and cerebrovascular disease and no adverse effects have been reported till now. Lam et al. reported that, TSA was detected in blood within 5 minutes after intraperitoneal injection and also detected in the brain 5 minutes after injection, showing that the drug was able to penetrate the blood brain barrier. The level in the brain reached the peak at 60 minutes and decreased slowly over several hours, and was undetectable after 8 hours, showing the drug was able to affect the brain [10]. In our previous study, we found that 25 mg/kg TSA was an optimal concentration. A dose of 25 mg/kg TSA or 1 ml/kg PBS including 1% dimethyl sulfoxide (DMSO) was given at 3 d before MCAO (once a day for 2 consecutive days with the last injection 2 h before MCAO), 10 min after MCAO, 0 h after reperfusion, and 2 h after reperfusion. The results showed that 25 mg/kg TSA reduced infarct size relative to the vehicle+I/R group at all points in time, of which 10 min after MCAO was the most significant [15]. So we chose to administer 25 mg/kg TSA 10 min after MCAO in the present experiments.

Focal cerebral I/R injury produces core infarct tissue that is severely compromised and may not be repairable. The surrounding tissue, the peri-infarct region, may be subjected to further damage through activation of secondary inflammatory and neurodegenerative cascades [20]. Kim et al. and Phillips et al. reported that inflammatory and immunological reactions are involved in the pathogenesis of cerebral ischemia following blood reperfusion to the surrounding tissue [21], [22]. In this way, the inhibition of inflammatory responses, especially inhibition of inflammatory cytokine activities at the early stage of ischemia, may constitute an attractive therapeutic strategy. Our results demonstrated that I/R can lead to neurological dysfunction, brain edema, brain infarction, and neutrophil infiltration. However, these changes were attenuated by TSA. This indicates that the protective effect of TSA may be associated with inhibition of inflammation.

MIF is an upstream regulator of inflammatory immune processes [23]. After activating NF-κB, MIF can induce the production of subsequent cytokines [8]. A recent study has demonstrated that MIF KO mice with I/R had reduced expression of various inflammatory cytokines and mediators [24]. Wang et al. showed that MIF expression is increased at the transcriptional level in human stroke patients and in animal models of focal ischemia [9]. This suggests that inhibition of MIF may represent a suitable treatment strategy for ischemic stroke. Our results demonstrated that TSA could inhibit MIF expression, NF-κB activity and the release of TNF-α and IL-6 in the ischemic cortex induced by cerebral I/R injury at different points in time. This provided the evidence that MIF associated with the NF-κB activation pathway plays an important role in the injury processes after stroke. Inhibition of the up-regulation of MIF, NF-κB, TNF-α and IL-6 during the early stage brings great benefits to the organism.

Inácio et al. showed that, 5 days after surgery, MIF increases around the infarct core in the permanent middle cerebral artery occlusion in rats, where it is located to neurons and astrocytes [25]. Our results showed that, in the cerebral cortex, MIF is present almost all located in neurons and hardly any located in astrocytes. Following MCAO, MIF significantly increased around the infarct core in the vehicle+I/R group and significantly decreased in the TSA+I/R group at 24 hours after reperfusion.

As a regulator of death and survival proteins, NF-κB plays an important role in neuron survival within the central nervous system. Persistent activation of NF-κB renders neurons vulnerable to ischemic insult [26], [27]. Our results demonstrate that NF-κB binding activity is increased in the vehicle+I/R group 24 hours after focal cerebral I/R and significantly lower in the TSA treatment group than in the vehicle+I/R group. Based on these results, treatment with TSA can be said to hamper the movement of NF-κB from the cytoplasm to the nucleus. Moreover, Jang et al. reported that TSA inhibited NF-κB-DNA complex, NF-κB binding activity, and the phosphorylation of IκBα in RAW 264.7 cells stimulated with LPS [28]. Their reports are consistent with our study and also suggest that TSA may inhibit IκBα degradation and NF-κB activation via suppression of the NIK–IKK pathway as well as the MAPKs (p38, ERK1/2, and JNK) pathway. Whether TSA inhibits NF-κB activation in the brain also via these pathways, it is still unclear and worth of further exploration.

Collectively, our results suggest that TSA has a protective effect on the brain I/R injury and that this effect takes place partly through its anti-inflammatory effect, which is associated with down-regulation of MIF in neurons.

## Materials and Methods

### Chemicals

TSA (purity 99%, HPLC) was obtained from Sciphar Biotechnology Co (Shaanxi, China). TSA was dissolved in phosphate-buffered saline (PBS) including 1% DMSO before the experiments. 2,3,5-triphenyltetrazolium chloride (TTC) was purchased from Sigma-Aldrich Co. (St. Louis, MO, U.S.). A determination of MPO kit was purchased from the Jiancheng Bioengineering Institute (Nanjing, China). Enzyme-linked immunosorbent assay (ELISA) kits were purchased from R&D Systems (Minneapolis, MN, U.S). MIF, NF-κB p65, and β-actin antibodies were purchased from Santa Cruz Biotechnology, Inc. (Santa Cruz, CA, U.S.). NeuN antibody was obtained from Merck Millipore (MA, U.S.). GFAP antibody was obtained from Boster Bio-engineering Co. Ltd. (Wuhan, China).

### Animals

Adult male Sprague-Dawley rats weighing 250±20 g (n = 132), age 90±4 d, were bred and held in the Experimental Animal Center of Chongqing Medical University. The protocol was approved by the institutional animal care and use committee and the local experimental ethics committee. All rats were allowed free access to food and water before the operation under optimal conditions (12/12 hours light/dark with humidity 60±5%, 22±3°C).

### Rat Model of Transient Focal Cerebral Ischemia

Transient cerebral ischemia was induced by MCAO in rats as described in detail in our previously study [29], [30]. The middle cerebral artery was occluded by insertion of a nylon filament (diameter 0.24–0.28 mm). After 2 hours of ischemia, the nylon filament was carefully pulled out to establish reperfusion. Rats that did not show neurological deficits immediately after reperfusion (neurological score <1) were excluded from the study. Rats that showed neurological deficits immediately after reperfusion (neurological score >0) but were found to be experiencing skull base or subarachnoid hemorrhage were also excluded from the study. The total elimination rate was 14/132. Sham-operated animals underwent the same surgical procedures without occlusion of the common carotid arteries.

### Groups and Drug Administration

One hundred and thirty-two rats were divided into 3 groups randomly: sham group, vehicle+I/R group, 25 mg/kg TSA+I/R group (n = 44 for each group). TSA was injected intraperitoneally 10 minutes after MCAO. Our previously study demonstrated that 25 mg/kg TSA 10 minutes after MCAO had significant protective effects [15]. A vehicle of 1 ml/kg PBS including 1% DMSO was given.

### Assessment of Neurological Deficits

Neurological testing of the vehicle- and drug-treated groups was carried out after 2 hours of MCAO by one examiner blinded to the experimental groups according to the method described by Longa et al. [18]. Neurological findings were scored on a five-point scale: 0, no neurological deficits; 1, failure to extend right forepaw fully; 2, circling to the right; 3, falling to the right; and 4, inability to walk spontaneously combined with depressed levels of consciousness.

### Measurement of Brain Water Content

Samples from all three groups (n = 6 for each group) were used for assessment. The rats were killed with chloral hydrate anesthesia 6 hours after reperfusion, and the brains were rapidly removed and dissected. Brain samples from the ischemic hemisphere were immediately weighed on an electronic balance and then dried in an oven at 100°C for 24 hours to determine the dry weight. Brain water content was calculated as follows: (wet weight − dry weight)/wet weight×100%.

### Measurement of Infarct Volume in the Brain

Samples from all three groups (n = 6 for each group) were used for analysis. At 6 hours after reperfusion, rats were killed and their brains were quickly removed and frozen at −20°C for 15 minutes. Coronal brain sections (2 mm thick) were stained with 2% TTC at 37°C for 20 minutes followed by fixation with 4% paraformaldehyde. The staining images were recorded using a digital camera (Canon Oxus 950IS) and then quantified using an Image J (ver 1.37c, NIH). To compensate for the effects of brain edema, the corrected volume was calculated using the following equation: Percentage hemisphere lesion volume (%HLV)  =  {[total infarct volume − (left hemisphere volume − right hemisphere volume)]/right hemisphere volume} ×100%. Infarct volume measurements were carried out by an investigator blinded to the treatment groups.

At the 1 hour, 3 hour, 6 hour, and 24 hour marks following sham or injury, rats were killed for sample collection (n = 5–8 for each point in time). For MPO activity assay, ELISA, Western blot, and EMSA analyses, ischemia cortex tissue was rapidly taken from fresh brains and immediately stored in liquid nitrogen.

### MPO Assay

The accumulation of neutrophils in the ischemia cerebral cortex was assessed by measuring MPO activity according to the manufacturer’s instructions. Briefly, the brain tissue samples obtained 6 hours and 24 hours after reperfusion were homogenized in cool normal saline (brain tissue: normal saline = 1∶10). MPO activity was measured with a spectrophotometer at 460 nm.

### Cytokine ELISA

Brain tissue homogenate was prepared using samples taken at all four points in time. Levels of MIF, TNF-α and IL-6 were determined using commercially available ELISA kits according to the manufacturer’s instructions.

### Western Blot Analysis

The ischemic cortex was harvested for the assay of protein expressions 24 hours after reperfusion. Western blotting was performed as described previously [15], [31]. MIF antibody (diluted to 1∶500), NF-κB p65 antibody (diluted to 1∶500), and β-actin (diluted to 1∶1000) were used as primary antibodies. Blots were subjected to gel formatter (BIO-RAD) and quantified through Quantity One analysis. β-Actin was used as an internal loading control.

### Nuclear Protein Extraction and EMSA

Nuclear proteins from cortical tissue were extracted and quantified as described [32], [33]. In brief, frozen brain samples were homogenized in 0.8 ml ice-cold buffer A, composed of 10 mmol/L HEPES (pH 7.9), 10 mmol/L KCl, 2 mmol/L MgCl_2_, 0.1 mmol/L EDTA,1 mmol/L dithiothreitol (DTT), and 0.5 mmol/L phenylmethylsulfonyl fluoride (PMSF) (all from Sigma Chemical Co.). The homogenates were incubated on ice for 25 minutes and vortexed for 30 seconds after addition of 50 µL 10% NP-40 (Sigma Chemical Co.). The mixture was then centrifuged for 10 minutes at 5000 g at 4°C. The pellet was suspended in 200 µL ice-cold buffer B composed of 50 mmol/L HEPES (pH 7.9), 400 mM NaCl, 50 mmol/L KCl, 0.1 mmol/L EDTA, 1 mmol/L DTT, and 0.5 mmol/L PMSF, and 25% (v/v) glycerol and incubated on ice 30 minutes with frequent mixing. After centrifugation (12,000 ×g, 4°C) for 15 minutes, the supernatants containing nuclear proteins were collected and stored at *−*80°C for further analysis. Protein concentration was determined using a bicinchoninic acid assay kit with bovine serum albumin as the standard (Pierce Biochemicals).

EMSA was performed using a commercial kit (Gel Shift Assay System; Promega, Madison, WI, U.S.) using methods described in detail elsewhere [32], [34]. The NF-κB consensus oligonucleotide probe (5′-AGTTGAGGGGACTTTCCCAGGC-3′) was end-labeled with [*γ*-^32^P] ATP (Free Biotech., Beijing, China) with T4-polynucleotide kinase. Nuclear protein (15 µg) was preincubated in a total volume of 9 µL in a binding buffer, consisting of 10 mmol Tris-HCl (pH 7.5), 20 mmol NaCl, 1 mmol MgCl_2_, 0.5 mmol DTT, 0.5 mmol EDTA, 4% glycerol, and 0.05 mg/mL poly-(deoxyinosinicdeoxycytidylic acid) for 15 minutes at room temperature. After addition of the 1 µL ^32^P-labled oligonucleotide probe, the incubation was continued for 20 minutes at room temperature. The reaction was stopped by adding 1 µL of gel loading buffer. The mixture was subjected to nondenaturing 4% polyacrylamide gel electrophoresis in a TBE buffer (Tris-borate-EDTA). The gel was vacuum-dried and exposed to X-ray film (Fuji Hyperfilm, Tokyo, Japan) at −80°C with an intensifying screen. Levels of NF-κB DNA binding activity were quantified by computer-assisted densitometric analysis.

### Immunofluorescence Staining

The samples used for histological assessment in our previous study were also used for immunofluorescence staining here. Briefly, sections were incubated for 15 min in 1% Triton X-100 to disrupt the cell membrane after the removal of wax and benzene. Brain tissues were microwaved for 10 min for antigen retrieval, and then allowed to cool down. They were then placed in PBS and blocked with normal goat serum at 37°C for 30 min. Mouse anti-GFAP and anti-NeuN and rabbit anti-MIF were used as primary antibodies. Sections were incubated in primary antibody (anti-GFAP, 1∶200; anti-NeuN, 1∶200; anti-MIF, 1∶100) overnight at 4°C, followed by reaction for 1 h at 37°C with FITC-conjugated goat anti-rabbit IgG antibody (1∶100) and TRITC-conjugated goat anti-mouse IgG antibody (1∶200). After washing, the sections were cover slipped with glycerin jelly. Images were captured and digitized using a Leica laser scanning confocal microscope (Leica TCS-SP2).

### Statistical Analysis

All data are expressed as mean ± S.E.M. The differences between various groups were analyzed by one-way analysis of variance (ANOVA) followed by multiple comparison tests as post hoc comparison. The neurological deficit scores were analyzed by Mann-Whitney U test. A level of *P*<0.05 was considered to be statistically significant. Statistical software package SPSS17.0 was used.
